# Acute Kidney Injury in Cardiac Surgery Patients: Role of Glomerular Filtration Rate and Fat-Free Mass

**DOI:** 10.15388/Amed.2021.28.1.22

**Published:** 2021-05-17

**Authors:** Elija Januškevičiūtė, Vaidas Vicka, Justina Krauklytė, Alvita Vickienė, Donata Ringaitienė, Mindaugas Šerpytis, Jūratė Šipylaitė

**Affiliations:** Faculty of Medicine, Vilnius University, Vilnius, Lithuania; Department of Anesthesiology and Intensive Care, Institute of Clinical Medicine, Faculty of Medicine, Vilnius University, Vilnius, Lithuania; Faculty of Medicine, Vilnius University, Vilnius, Lithuania; Department of Nephrology and Kidney Transplantation, Centre of Nephrology, Vilnius University Hospital Santaros Klinikos, Vilnius, Lithuania; Department of Anesthesiology and Intensive Care, Institute of Clinical Medicine, Faculty of Medicine, Vilnius University, Vilnius, Lithuania; Department of Anesthesiology and Intensive Care, Institute of Clinical Medicine, Faculty of Medicine, Vilnius University, Vilnius, Lithuania; Department of Anesthesiology and Intensive Care, Institute of Clinical Medicine, Faculty of Medicine, Vilnius University, Vilnius, Lithuania

**Keywords:** acute kidney injury, eGFR, fat-free mass

## Abstract

**Summary. Background.:**

eGFR (estimated glomerular filtration rate) formulas may be inaccurate in overweight cardiac surgery patients, overestimating the kidney reserve. The aim of this study was to modify the eGFR formulas and to determine whether the modified eGFR is a more accurate predictor of acute kidney injury (AKI).

**Materials and methods:**

The patients were assigned into 4 BMI groups as follows: normal weight (18.5– 25 kg/m^2^), pre-obesity (25–30 kg/m^2^), class I obese (30–35 kg/m^2^), class II and III obese (≥35 kg/m^2^). Cockcroft– Gault (CG) eGFR formula was modified by using the fat-free mass (FFM) derived from bioelectrical impedance. ROC-AUC curves were analyzed to identify the accuracy of the eGFR formulas (CG, CG modified with FFM, Mayo Clinic Quadratic equation, CKD-EPI, MDRD) to predict the AKI in each group.

**Results:**

Although all of the used equations showed similar predictive power in the normal weight and overweight category, Mayo formula had the highest AUC in predicting the occurrence of AKI (ROC-AUC 0.717 and 0.624, p<0.05). However, in the group of patients with class I obesity, only the CG formula modified with a fat-free mass appeared to be predictive of postoperative AKI (ROC-AUC 0.631 p<0.05). None of the equations were accurate in the group of BMI (>35 kg/m^2^).

**Conclusions:**

eGFR is a poor predictor of AKI, especially in the obese patients undergoing cardiac surgery. The only equation with a moderate predictive power for the class I obese patients was the CG formula modified with the fat-free mass.

## Abstract (lt)

**Ūminio inkstų funkcijos pažeidimo vertinimas širdies chirurgijoje: glomerulų filtracijos greičio ir liesosios kūno masės reikšmė:**

**Santrauka. *****Įžanga:*******Glomerulų filtracijos greičio (GFG) nustatymo formulės gali būti netikslios, kai naudojamos antsvorį turintiems pacientams – inkstų rezervas ir funkcija yra įvertinami per gerai. Šio tyrimo tikslas – modifikuoti GFG nustatymo formules įtraukiant liesąją kūno masę ir nustatyti, ar taip apskaičiuotas GFG yra geresnis pooperacinio ūminio inkstų pažeidimo (ŪIP) prediktorius pacientams, kuriems atliekamos širdies operacijos.

***Medžiagos ir metodai:*******Pacientai buvo suskirstyti į 4 grupes, atsižvelgiant į kūno masės indeksą (KMI): normalaus svorio (18,5–25 kg/m^2^), turintys antsvorį (25–30 kg/m^2^), turintys I laipsnio nutukimą (30–35 kg/m^2^), turintys II ar III laipsnio nutukimą (≥ 35 kg/m^2^). GFG skaičiuoti buvo naudotos šios formulės: „*Cockroft-Gault*“ (CG), „*Mayo Clinic Quadratic*“, CKD-EPI, MDRD. CG formulė buvo modifikuota naudojant liesąją kūno masę, nustatytą bioimpedanso analizės metu, ja keičiant bendrąją kūno masę. Pooperacinis ūminis inkstų pažeidimas (ŪIP) buvo apibrėžtas naudojant „*Society of Thoracic Surgeons*“ kriterijus. Formulių jautrumui numatant pooperacinį ŪIP skirtingose grupėse nustatyti ir palyginti naudota ROC-AUC analizė.

***Rezultatai:*******Normalaus kūno svorio ir antsvorio kategorijose daugumos formulių tikslumas, prognozuojant ŪIP atsiradimą, buvo panašus. *Mayo* formulės plotas po kreive buvo didžiausias (ROC-AUC 0,717 ir 0,624, p < 0,05). Pacientams, turintiems I laipsnio nutukimą, vienintelė GFG formulė, patikimai prognozuojanti ŪIP, buvo pagal liesąją kūno masę apskaičiuota CG formulė (ROC-AUC 0,631, p < 0,05). Nė viena iš lygčių nebuvo tiksli paskutinėje KMI grupėje (> 35 kg/m^2^).

***Išvados: ***Vienintelė GFG apskaičiavimo formulė, tinkama prognozuoti ŪIP po širdies operacijų I laipsnio nutukimą turintiems pacientams, buvo pagal liesąją kūno masę apskaičiuota CG formulė. GFG yra prastas prognostinis ŪIP po širdies operacijų rodiklis II ir III laipsnio nutukimą turintiems pacientams.

**Raktažodžiai: **ūminis inkstų pažeidimas, GFG, aktyvioji kūno masė.

## Introduction

Acute kidney injury (AKI) is one of the most common complications of cardiac surgery. Every year, there are more than 2 million cardiac surgeries performed and the incidence of AKI after cardiac surgery varies from 5 to 42 percent [1]. Moreover, severe AKI is associated with higher perioperative mortality and prolonged stay in intensive care unit and hospital [2]. Cardiac surgery associated AKI is related to various risk factors: age, hypertension, hyperlipidemia, peripheral vascular disease, diabetes mellitus, chronic obstructive pulmonary disease and obesity; all of them resulting in a decreased preoperative kidney reserve [3,4].

Over the years, one of the ways to predict a postoperative AKI was measuring the preoperative creatinine concentration and employing it to estimate the glomerular filtration rate (eGFR). Serum creatinine is not specifically related to renal dysfunction, but is also affected by age, muscle mass, gender, ethnicity and diet. Furthermore, the concentration of the creatinine is in the normal range even if there is a notable decrease in renal reserve [5]. Therefore, eGFR has been proposed as a more reliable measure than serum creatinine in identifying and assessing the renal function in perioperative period [6–8]. To this day, there are multiple formulas for calculating eGFR, which include Cockcroft–Gault (CG), Modification of Diet in Renal Disease formula (MDRD), Chronic Kidney Disease Epidemiology Collaboration (CKD-EPI) equation and Mayo Clinic Quadratic equation (Mayo). The newest equations, such as MDRD or CKD-EPI do not include patient’s weight into the calculations. The main reason for that is the low effect of weight on the result, since net weight of the patient may be increased due to factors not related to the kidney function. The older Cockcroft–Gault formula is the only one that includes body weight, nowadays enhanced by indexing it to the patient’s body surface. However, both the older and the new equations do not provide a satisfactory accuracy in predicting the postoperative kidney function, especially for the obese patients.

Bioelectrical impedance analysis (BIA) is a method that serves to evaluate the body’s composition and to discriminate the exact proportion of water, fat mass and fat-free mass [9]. The principle of BIA is to measure the different impedance of the tissues. A small voltage alternating current is transmitted through the body, showing the impedance which can be measured. The measured impedance is then compared to the population-based reference and transformed into mass and volume units accordingly. The reference values are specific to the ethnicity, age, gender and BMI of patients. The aim of this study was to modify the CG formula by using the fat-free mass derived from BIA instead of the whole body mass and to determine whether the modified eGFR formula is a more accurate predictor of acute kidney injury after cardiac surgery, focusing on the overweight patients.

## Materials and methods

*Study population.* This was a retrospective study of patients who underwent elective cardiac surgery in a tertiary referral university hospital in a period of one year. Ethical approval was obtained from the Regional Research Ethics Committee and all patients provided written and informed consent to participate in the study. Inclusion criteria were as follows: primary elective cardiac surgery with median sternotomy, 18 years or older, cooperative and able to provide written informed consent. The following exclusion criteria were applied: patients with implanted defibrillators and major amputations. General preoperative characteristics were evaluated: age, sex, weight, body mass index (BMI), European System for Cardiac Operative Risk Evaluation (EuroSCORE) II, comorbidities and type of surgery. Details of procedure, such as usage and length of cardiopulmonary bypass (CPB), aorta clamp time and duration of surgery as well as preoperative and postoperative serum creatinine levels were collected. The patients were assigned into 4 BMI groups as follows: group 1 (BMI <25 kg/m^2^), group 2 (BMI 25–30 kg/m^2^), group 3 (30–35 kg/m^2^), group 4 (≥35 kg/m^2^) according to the WHO classification. 

*Evaluation of kidney function*. The patients’ baseline serum creatinine (sCr) value was collected and GFR was estimated using the MDRD, CKD-EPI, Mayo and CG equations. Considering the idea that total body weight might lead to misinterpretation of actual renal function, CG equation was modified by replacing the whole body mass with the fat-free mass derived from the BIA (CGFFM). Furthermore, the CG formula was adjusted to the body surface. The postoperative AKI was defined by KDIGO creatinine change definitions, considering AKI as a 0.3 mg/dl (≥26.5 mol/l) sCr increase from baseline within 48 hours of surgery, or a 50% sCr increase from baseline within 7 days of surgery [10]. Since urine output changes are not specific, especially during cardiac surgery with cardiopulmonary bypass (CPB), as well as due to medications, such as diuretics and mannitol, the urine output criteria arm was omitted. [11]

*Bioelectrical impedance analysis.* BIA was performed to all the patients on the day before the surgery. InBody 72 S10 device (Biospace, Seoul, Korea) was used following the instructions suggested by the European Society for Clinical Nutrition and Metabolism: in a supine position with arms abducted 15° from the trunk and legs spread apart at shoulder width. The analysis was performed using eight electrodes placed on both hands (on thumb and middle finger) and between the patient’s anklebones and heels [9]. The measurements obtained were: intracellular water, extracellular water, total body water and fat-free mass. These values were derived from the reference used in the machine, different for ethnicity, age, gender and BMI.

*Statistical Analysis.*****Statistical analyses were carried out by the SPSS statistical software package version 26.0 (IBM/SPSS, Inc., Chicago, IL). Baseline characteristics were described using descriptive statistics. Categorical variables were reported as an absolute number (*n*) and a relative frequency (%), and continuous variables were expressed as a median (interquartile range) or as a mean (± SD), depending on the normality of the distribution. To determine the distribution of each variable, the Kolmogorov–Smirnov test was used. Test values of p<0.05 were considered statistically significant. 

Receiver operating characteristics (ROC) curves were analyzed to identify the accuracy of the eGFR ability to predict the AKI. All the calculations were performed in different categories of BMI.

## Results

*Baseline characteristics of study participants. *476 patients were enrolled, 67.2% of them were men. The age median was 65.0 [58.0–73.0] years. Most of the patients underwent coronary artery bypass (CABG) surgery (58.6%) and had a low operative risk, with a median EuroScore II of 1.69 [1.05– 2.49]. 77.3% of the surgeries involved on-pump technique, with a median CPB time of 116 [93–154] minutes. The most common comorbidities in the present population were hypertension (79%), diabetes (22.5%), and peripheral vascular disease (14.7%). 90.7% of the patients were classified as New York Heart Association class II to III, and 19.5% of the patients were current smokers (Table 1). 

**Table 1.** Baseline Characteristics of the Patients

N(%)/Median (IQR)/ Mean (±SD)					
BMI categories (kg/m^2^)	18.5–24.9	25–29.9	30–34.9	>35	Total
**Patients**					
Amount	90 (18.9)	226 (47.5)	108 (22.7)	52 (10.9)	467 (100)
Sex, male	72 (80.0)	148 (65.5)	71 (65.7)	29 (55.8)	320 (67.2)
Age, y	65.0 [56.8–73.0]	65.5 [58.0–73.0]	65.0 [58.0–74.0]	64.5 [57.3–70.8]	65.0 [58.0–73.0]
**Preoperative data**					
EuroSCORE II, %	1.64 [1.06–3.21]	1.61 [1.05–2.60]	1.73 [0.99–2.20]	1.77 [1.15–2.26]	1.69 [1.05–2.49]
Diabetes	8 (8.9)	44 (19.5)	33 (30.6)	22 (42.3)	107 (22.5)
Diabetes on insulin	3 (3.3)	14 (6.2)	16 (14.8)	13 (25.0)	46 (9.7)
Chronic kidney disease	3 (3.3)	6 (2.7)	3 (2.8)	4 (7.7)	16 (3.4)
Hypertension	61 (67.8)	183 (81.0)	87 (80.6)	45 (86.5)	376 (79)
Stroke	12 (13.3)	20 (8.8)	8 (7.4)	5 (9.6)	45 (9.5)
COPD	6 (6.7)	11 (4.9)	5 (4.6)	4 (7.7)	26 (5.5)
PVD	16 (17.8)	31 (13.7)	15 (13.9)	8 (15.4)	70 (14.7)
Oncological disease	8 (8.9)	12 (5.3)	7 (6.5)	7 (13.5)	34 (7.1)
MI <90 days	5 (5.6)	23 (10.2)	10 (9.3)	2 (3.8)	40 (8.4)
Smoking history	28 (31.1)	48 (21.2)	14 (13.0)	3 (5.8)	93 (19.5)
LVEF, %	55 [45–55]	55 [45–55]	55 [50–55]	55 [50–55]	55 [45–55]
NYHA class II-III	83 (92.2)	205 (90.7)	96 (88.9)	48 (92.3)	432 (90.7)
**Type of surgery**					
CABG	44 (48.9)	141 (62.4)	63 (58.3)	31 (59.6)	279 (58.6)
Valve surgery	56 (62.2)	96 (42.5)	41 (38.0)	26 (50.0)	219 (37.8)
Combined	4 (4.4)	5 (2.2)	7 (6.5)	1 (1.9)	17 (3.6)
**Details of the procedure**					
Use of CPB	74 (82.2)	169 (74.8)	85 (78.7)	40 (76.9)	368 (77.3)
CPB time, min	116 [97–166.3]	117 [92–147]	121 [95–154]	106 [91–159]	116 [93–154]
Aorta clamp time, min	75 [59–110]	72 [59–93]	77 [62–104]	70 [62–106]	74 [60–100]
Duration of surgery, min	210 [180–265]	200 [180–250]	210 [180–255]	210 [180–270]	210 [180–255]
**Body composition**					
BM, kg	68.5 [62.0–75.0]	80.0 [73.0–86.0]	93.0 [87.0–102.8]	110.0 [97.5–118.0]	82.0 [73.0–92.6]
BMI, kg/m^2^	23.4 [21.5–24.4]	27.5 [26.2–28.5]	32.4 [31.2–33.7]	37.1 [35.9–39.0]	28.1 [25.7–31.8]
Surface area, m^2^	1.8 (±0.16)	2.0 (±0.17)	2.1 (±0.17)	2.3 (±0.22)	2.0 (±0.22)
FFM, kg	53.8 (±8.89)	55.8 (±10.64)	58.4 (±11.82)	60.8 (±11.76)	56.6 (±10.92)
FFMI, kg/m^2^	18.0 (±1.80)	19.1 (±2.07)	20.1 (±2.55)	21.3 (±2.22)	19.4 (±2.35)
IBW, kg	67.7 [62.3–72.5]	65.9 [56.0–72.3]	65.9 [56.0–71.4]	64.1 [54.2–70.5]	65.9 [56.0–71.4]

NOTE. Data are presented as the median and range, mean and standard deviation or percentage. Abbreviations: IQR, interquartile range; COPD, chronic obstructive pulmonary disease; PVD, peripheral vascular disease; MI, myocardial infarction; LVEF, left ventricular ejection fraction; NYHA, New York Heart Association; CABG, coronary artery bypass grafting; CPB, cardiopulmonary bypass; BM, body mass; BMI, body mass index; FFM, fat-free mass; FFMI, fat-free mass index; IBW, ideal body weight.

*Evaluation of body composition. *After assigning patients into 4 BMI groups, capacity of each group was determined: 90 (18.9%) were in group 1, 226 (47.5%) were in group 2, 108 (22.7%) were in group 3, and 52 (10.9%) were in group 4. Evaluation of body composition parameters demonstrated following findings: BIA derived fat-free mass showed a slight increase from group 1 (53.8±8.89 kg) to group 4 (60.8±11.76 kg) leading to the fat-free mass index (FFMI) increase from 18.0±1.80 kg/m^2^ to 21.3±2.22 kg/m^2^.

*Preoperative kidney reserve and postoperative kidney function. *Preoperative measured serum creatinine median was 0.92 [0.52–2.35] mg/dL. After applying MDRD, CKD-EPI and CG equations, the mean measured eGFR varied accordingly: MDRD 74.75±19.74 ml/min/1,73m^2^, CKD-EPI 78.49 [64.09–91.79] ml/min/1,73m^2^, CG 86.42 [67.14–109.45] ml/min/1,73m^2^ and Mayo 92.49±21.68 ml/min/1,73m^2^ (Table 2). When comparing the GFR across the weight groups it was evident, that the GFR measured by MDRD and CKD-EPI was decreasing as the weight increased, reaching the lowest point in group 3 (68.38 (±17.63) and 71.18 [58.97–86.53] ml/min/1,73m^2^). Oppositely, the values of CG derived GFR were highest in the group 3 (105.72 [85.03–131.39] ml/min/1,73m^2^). After applying modifications to the Cockcroft–Gault equation, CG-FFM derived eGFR provided the lowest value from all the equations – 58.46 [43.82–76.11] ml/min/1,73m^2^. This relationship was evident across all the weight groups (Table 2). 16 patients were diagnosed with CKD before the study, with no statistically relevant distribution across the BMI groups. 

A total of 138 patients developed acute kidney injury after cardiovascular surgery (29%). The majority of patients (20.2%) were diagnosed with stage 1 AKI, 4% and 4.8% – Stage 2 and Stage 3 AKI accordingly.

**Table 2.** Evaluation of preoperative kidney reserve

Mean (±SD)/ Median (IQR)					
BMI categories	18.5–24.9	25–29.9	30–34.9	>35	Total
Serum creatinine, mg/dL	0.92 [0.80–1.10]	0.99 [0.81–1.06]	0.96 [0.82–1.05]	0.94 [0.82–1.16]	0.92 [0.52–2.35]
eGFR-MDRD, ml/min/1,73m^2^	77.73 ±22.59)	74.52 ±19.60)	75.81 ±17.94)	68.38 ±17.63)	74.75 ±19.74)
eGFR-CKDEPI, ml/min/1,73m^2^	83.15 [67.32–94.04]	77.60 [63.11–91.58]	77.87 [66.92–92.15]	71.18 [58.97–86.53]	78.49 [64.09–91.79]
eGFR-CG, ml/min/1,73m^2^	75.33 [55.44–96.93]	81.75 [63.57–103.21]	98.34 [81.93–120.72]	105.72 [85.03–131.39]	86.42 [67.14–109.45]
eGFR-CG-FFM, ml/min/1,73m^2^	58.05 [41.42–76.44]	57.09 [42.55–74.41]	61.62 [47.35–78.64]	61.27 [42.71–74.70]	58.46 [43.82–76.11]
eGFR-Mayo, ml/min/1,73m^2^	95.06 ±26.28)	92.06 ±21.04)	93.74 ±18.91)	87.31 ±20.67)	92.49 ±21.68)

NOTE. Data are presented as the mean and standard deviation or median and range. Abbreviations: eGFR, estimated glomerular filtration rate; CG, Cockcroft–Gault; MDRD, Modification of Diet in Renal Disease; CKD-EPI, Chronic Kidney Disease Epidemiology Collaboration; Mayo, Mayo Clinic Quadratic.

**Fig. 1.**
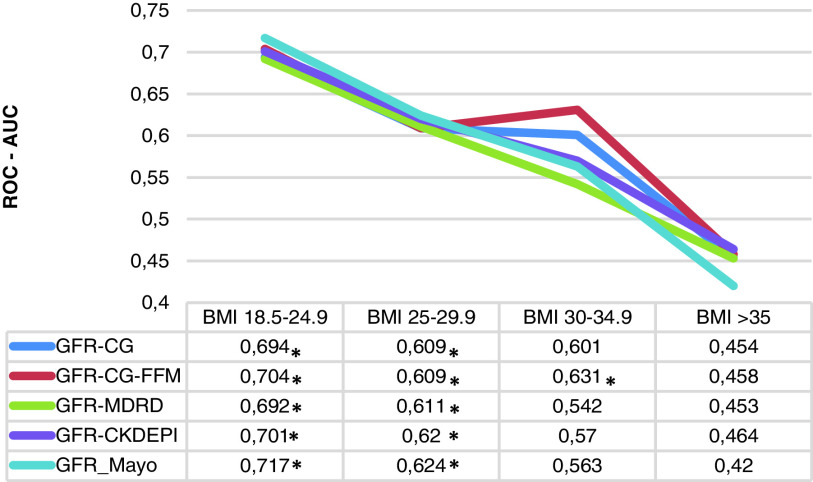
Predictive power of eGFR for AKI in different BMI categories

Abbreviations: AKI, acute kidney injury, BMI, body mass index; eGFR, estimated glomerular filtration rate; CG, Cockcroft–Gault; MDRD, Modification of Diet in Renal Disease; CKD-EPI, Chronic Kidney Disease Epidemiology Collaboration.

*Predictive power of eGFR. *eGFR potency to predict postoperative AKI was evaluated by area under the receiver operating characteristic curve (ROC-AUC) analysis (Figure 1). Results revealed descending tendency of eGFR prediction accuracy as the patients’ weight increase. Although all of the used equations showed similar predictive power in the first BMI category, Mayo formula had the highest AUC in predicting the occurrence of AKI (ROC-AUC 0.717, p<0.05). A similar pattern was observed in the group of overweight patients, with values for the ROC-AUC curves between 0.609 and 0.624, p<0.05 (eGRF calculated using the Mayo equation showed the best AKI prediction capacity). However, in the group of patients with class I obesity, only the CG-FFM appeared to be able to predict postoperative AKI (ROC-AUC 0.631, p<0.05). None of the equations demonstrated statistically significant prediction power in the 4 group (class II and III obesity).

* p-value less than 0.05

## Discussion

The results of this retrospective study of 476 patients who underwent cardiac surgery showed that the estimated glomerular filtration rate is a poor predictor of acute kidney injury, especially in the obese patient group. The only equation with a moderate predictive power for the obese patients was developed from the CG one, exchanging the whole body mass with a bioelectrical impedance analysis derived fat-free mass or ideal body weight.

The main finding of the study is a poor accuracy of eGFR formulas in predicting the AKI after cardiac surgery, especially for the overweight and obese patients. When BMI is between 18.5 and 29.9 kg/m^2^, all equations perform similarly with a small advantage of Mayo Quadratic formula. However, as the BMI gets higher all of the formulas perform poorly. This discrepancy can be explained by the straightforwardness of the BMI – it is well known that the whole mass of the body is not evenly distributed in different individuals. In this particular case the extracellular compartment was enlarged with excess fluid and the cellular compartment decreased because of lower than expected muscle mass. Fluid accumulation and decreased muscle mass are key features in chronic congestive heart failure. These pathological changes result in lower concentration of creatinine – both because of the dilution and production, and, consequently, a higher than expected eGFR calculated by the CG formula. Having these results in mind it is obvious that using a whole body weight for estimating the GFR is of questionable benefit, which has already been described in previous research when linking it to the AKI. The accuracy of the weight-based CG formula can be to some extent increased by replacing the whole body weight with a fat-free mass weight, reaching the AUC of 0.63. On the other hand, there is a strong relationship reported between the body mass and AKI after cardiac surgery. One study, completed in 2014, investigated the association between BMI and AKI incidence in 445 patients who underwent cardiac surgery, and stated that a greater BMI was associated with increased odds of AKI incidence [12]. Another retrospective study decided to classify the BMI of patients undergoing cardiac surgery after cardiopulmonary bypass into normal, overweight obesity class I, obesity class II, and obesity class III, based on the WHO definition for overweight and obesity. The results revealed that amongst the BMI groups, the obese group (BMI > 40) had a significantly greater chance of developing AKI after CPB than those in the lower BMI categories (12). These reports and the results of our study suggest a convoluted origins of the AKI for the obese patients undergoing the cardiac surgery (13). 

The following assumption can be made from the results of our study: estimated GFR is only one of the factors predicting the AKI in obese patients. The most plausible reason for that is the diminished kidney reserve, not yet indicated by the increase in serum creatinine. The likely pathophysiological mechanisms are the increase in adipose tissue together with insulin resistance, which leads to activation of the renin-angiotensin-aldosterone axis. As a result, there is an increase in stress in kidneys which is exaggerated by the inflammatory hormones and cytokines (14–16). Furthermore, obese patients have high kidney plasma flow and glomerular filtration rates, associated with glomerular capillary hypertension and causing occult structural changes in the kidneys. These mechanisms lead to hemodynamic injury to the glomerular capillary wall and glomerular hypertrophy. Moreover, increased insulin production, glomerular volume and capillary pressures have a huge further impact on the glomerular damage, creating a *circulus vitiosus* and leading to a chronic kidney disease. At this point the concentration of the creatinine is starting to increase, ergo the eGFR is decreased. Therefore, eGFR is suitable for kidney reserve measurement only in the advanced cases of kidney damage. This was not a case in our study population of low risk, mostly CABG patients. Despite that, even when correcting the CG formula with the fat-free mass, the modified eGFR did not reach a high accuracy when predicting the AKI in the obese patients, suggesting that more precise markers, derived from the before mentioned pathophysiological mechanisms, should be employed when estimating the risk of AKI in the obese patients. 

There are some limitations to the study. First of all, it is a retrospective observational study. The majority of study population are low risk, CABG patients, who are different from the high risk valve surgery patients, requiring a separate analysis. Secondly, the method employed to get an accurate value of the muscle mass was bioelectrical impedance analysis, which by itself has some limitations. The method is based on measurement of the impedance, and then using the impedance to get a muscle mass from the healthy population data. Even though the population used to set the references in the machine is concordant to the Caucasian population in our country it is not fully representative to the cardiac surgery patients. Furthermore, the selected outcome of the study – incidence of the AKI – is questionable, since the eGFR equations were created to stage the chronic kidney disease, not to predict the AKI, and are most accurate when eGFR is below 60 ml/min/ m^2^. It is difficult to estimate the bias of these inconsistencies, therefore the results of our study should be evaluated with care. 

## Conclusions

The estimated glomerular filtration rate is a poor predictor of acute kidney injury, especially in the obese patients undergoing cardiac surgery. The only equation with a moderate predictive power for the class I obese patients was the CG formula modified with the fat-free mass.

## Human and animal rights

All procedures performed in studies involving human participants were in accordance with the ethical standards of the institutional and/or national research committee at which the studies were conducted (IRB approval number 158200-12-561-162) and with the 1964 Helsinki declaration and its later amendments or comparable ethical standards.

## Conflict of interest

The authors have declared that no conflict of interest exists.

## Informed consent

Informed consent was obtained from all individual participants included in the study.
